# Porcine Alveolar Macrophage-like cells are pro-inflammatory Pulmonary Intravascular Macrophages that produce large titers of Porcine Reproductive and Respiratory Syndrome Virus

**DOI:** 10.1038/s41598-018-28234-y

**Published:** 2018-07-05

**Authors:** Elise Bordet, Pauline Maisonnasse, Patricia Renson, Edwige Bouguyon, Elisa Crisci, Mathieu Tiret, Delphyne Descamps, Cindy Bernelin-Cottet, Céline Urien, François Lefèvre, Luc Jouneau, Olivier Bourry, Jean-Jacques Leplat, Isabelle Schwartz-Cornil, Nicolas Bertho

**Affiliations:** 1 Virologie et Immunologie Moléculaires UR892, Institut National de la Recherche Agronomique, Domaine de Vilvert, 78352 Jouy-en-Josas, France; 2 Anses, Laboratoire de Ploufragan-Plouzane, Unité Virologie et Immunologie Porcines, Zoopôle, BP53, 22440 Ploufragan, France; 3 Université Bretagne Loire, Cité internationale, 1 place Paul Ricœur, CS 54417, 35044 Rennes, France; 4 Union des Groupements de Producteurs de Viande de Bretagne (UGPVB), 104 rue Eugène Pottier, 35065 Rennes, cedex France; 5 Génétique Animale et Biologie, Intégrative (GABI) UMR 1313, Institut National de la Recherche Agronomique, Domaine de Vilvert, 78352 Jouy-en-Josas, France; 6 Department of Population Health and Pathobiology, College of Veterinary Medicine, North Carolina State University, 1060 William Moore Drive, 27607 Raleigh, NC USA

## Abstract

Lung inflammation is frequently involved in respiratory conditions and it is strongly controlled by mononuclear phagocytes (MNP). We previously studied porcine lung MNP and described a new population of cells presenting all the features of alveolar macrophages (AM) except for their parenchymal location, that we named AM-like cells. Herein we showed that AM-like cells are macrophages phagocytosing blood-borne particles, in agreement with a pulmonary intravascular macrophages (PIM) identity. PIM have been described microscopically long time ago in species from the *Laurasiatheria* superorder such as bovine, swine, cats or cetaceans. We observed that PIM were more inflammatory than AM upon infection with the porcine reproductive and respiratory syndrome virus (PRRSV), a major swine pathogen. Moreover, whereas PRRSV was thought to mainly target AM, we observed that PIM were a major producer of virus. The PIM infection was more correlated with viremia *in vivo* than AM infection. Finally like AM, PIM-expressed genes were characteristic of an embryonic monocyte-derived macrophage population, whose turnover is independent of bone marrow-derived hematopoietic precursors. This last observation raised the interesting possibility that AM and PIM originate from the same lung precursor.

## Introduction

Respiratory infections are one of the major sources of disease in swine husbandry, leading to economic losses in the pig industry. Since the aetiology of respiratory diseases is multifactorial, the term porcine respiratory disease complex (PRDC) is often used^1^. One of the main pathogens at the root of PRDC is the Porcine Reproductive and Respiratory Syndrome Virus (PRRSV), an enveloped, positive-stranded RNA virus of the *Arteriviridae* family. Indeed, PRRSV presents long-term infections due to its capacities to alter the immune response, a property that facilitates bacterial and viral superinfections. PRRSV main cellular target is thought to be alveolar macrophages (AM), although virus is present in the blood and can be detected up to several months post infection in the secondary lymphoid organs (for review see^2^).

In order to further develop the pig as a biomedical model^3^, and to better deal with PRRSV infection as well as with PRDC, a better understanding of the immune environment of the pig lung is needed. We previously described dendritic cells (DC) and macrophages (MΦ) present in the porcine respiratory tract, such as conventional DC1 (cDC1), cDC2, monocyte-derived DC (moDC), and monocyte-derived MΦ (moMΦ) as well as AM^4^. As previously described in murine and human respiratory tracts, we observed that DC were present both in the parenchyma and in the alveoli, although at a lower level in the alveoli^5^. Surprisingly, we also observed the presence of macrophage-like cells with a phenotype highly similar to AM but that were not located in the alveoli. We thus named these parenchymal AM, AM-like cells. Despite thorough studies, this cell type has not been previously observed at steady state in mice^6^ or in non-human primates^7^. Interestingly 30 years ago, using electron microscopy and gold or iron oxide beads, a pulmonary macrophage population embedded in the endothelium wall of lung capillaries, called Pulmonary Intravascular Macrophages (PIM, for review see^8,9^) has been described in animals belonging to the *Laurasiatheria* superorder, for instance cattle, sheep, swine, horses, cats and odontoceti cetaceans^10–14^. No PIM were constitutively observed in monkeys, rabbits, mice or rats from the *Euarchontoglires* superorder^9,14^, which includes humans. PIM presented strong phagocytic capacities of blood born particles and their role in *Laurasiatheria* is thought to be similar to Kupffer cells in *Euarchontoglires*. Indeed PIM in the lung, as well as Kupffer cells in the liver are macrophages involved in the clearance of blood-borne particles^15^. Moreover, depletion studies of blood mononuclear phagocytes (MNP) in calves, horses and pigs have indicated that PIM might contribute to the acute lung inflammation induced by blood-borne pathogens by secreting pro-inflammatory cytokines such as TNFα, interleukin-8 (IL-8) and IL-6^16–20^. Despite these interesting early studies and the growing economic burden of PRDC in the porcine industry, PIM have rarely been considered in pig respiratory diseases during the last 10 years, most probably due to the difficulties to isolate and characterize them phenotypically and functionally.

Herein, we demonstrate in swine that the AM-like population represents pro-inflammatory PIM with the capacity to phagocytose blood-borne particles. We show that PIM present features of self-renewable, tissue-specific macrophages from embryonic-monocyte origin and that PIM are infected and produce large amount of PRRSV viral particles *in vitro* and *in vivo*.

## Results

### AM-like cells are Pulmonary Intravascular Macrophages (PIM)

**Figure 1:**
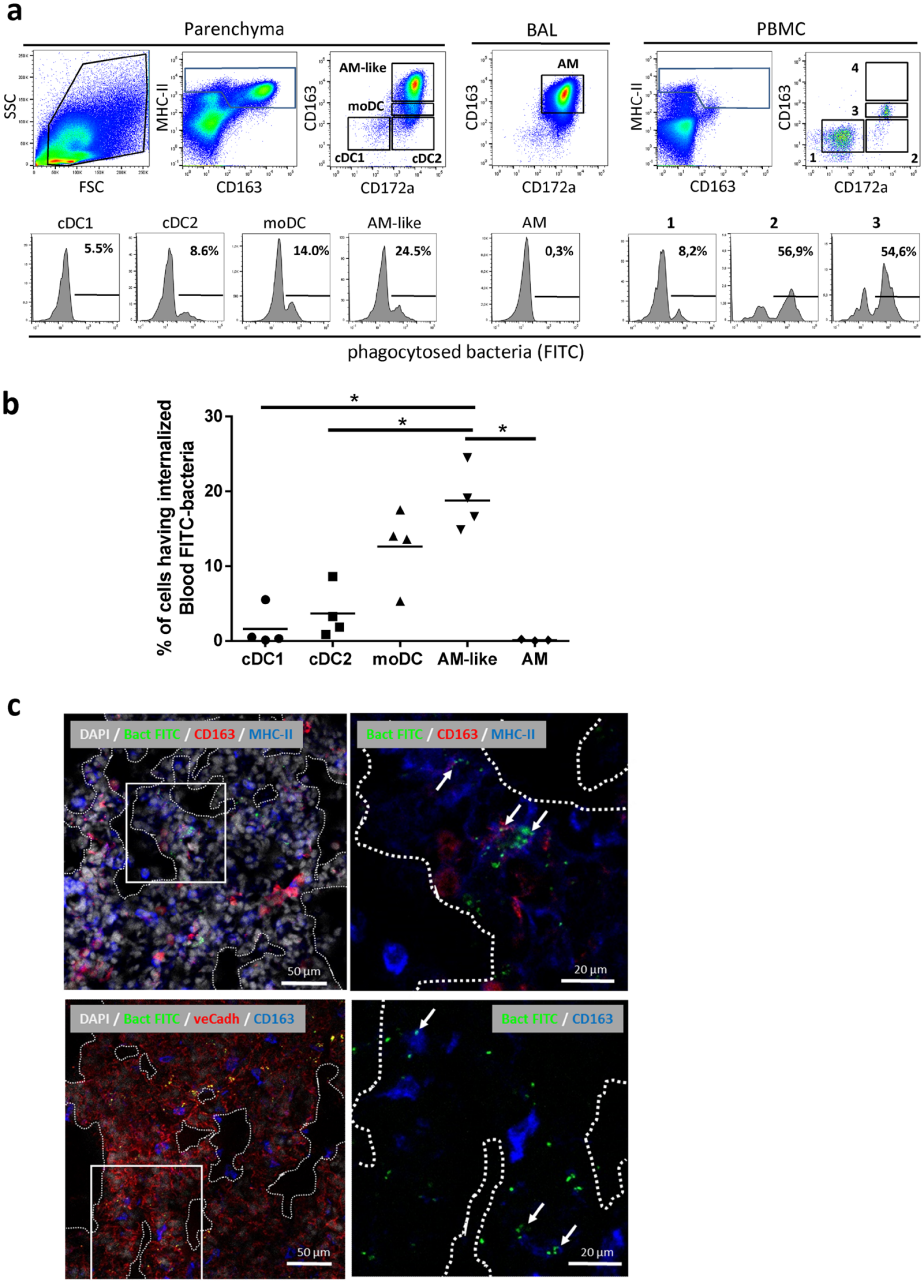
AM-like cells phagocytosed bloodborne bacteria. Inactivated FITC-labelled pseudomonas bacteria were injected in the jugular vein of anesthetized pigs. Ten minutes after, blood was collected on heparinized tubes and proceeded to Ficoll isolation of peripheral blood mononuclear cells (PBMC), animals were euthanized and cells from broncho-alveolar lavages and parenchyma were collected and stained for FACS analysis of DC and Mϕ. (**a**) Dot plot gating strategy of lung DC and Mϕ as previously described^4^ and histograms of phagocytosed bacteria (FITC) signal in the different gated populations. The PBMC gate 4 contained no event. One representative out of 4 independent experiments. (**b**) Plotting of the 4 independent experiments. Statistics: Mann-Whitney test *p < 0.05. (**c**) Confocal imaging of parenchyma from FITC-bacteria injected animals. Upper left, DAPI (white), FITC-bacteria (green), CD163 (red) and MHC-II (blue) staining. Upper right, magnification of the squared zone of the upper left picture. Arrows indicate PIM/AM-like cells (extra-alveolar CD163pos/MHC-IIpos) having internalized FITC-bacteria. Lower left, DAPI (white), FITC-bacteria (green), ve-Cadherin (red) and CD163 (blue) staining. Lower right, magnification of the squared zone of the lower left picture. Arrows indicate PIM/AM-like cells (extra-alveolar CD163pos) localized in ve-Cadherin rich area (lung capillaries) and having internalized FITC-bacteria. Alveoli are visualized by dashed lines. Images are representative of 3 independent experiments.

AM-like cells represented the second-most abundant population of lung MNP after AM, being 10 times less represented than AM, but at least 5 times more numerous than cDC1, cDC2 or moDC^5^. In a first description of AM-like cells, we demonstrated that these cells were not located in the alveoli, and that our broncho-alveolar lavage procedure avoids the contamination of the parenchymal cell preparation by AM^4^. However, we were uncertain whether AM-like cells were localized in the lung interstitium or on the endothelial wall, in contact with the vascular lumen as described for PIM. Since PIM have been described as highly phagocytic cells^20^, an experiment was devised in order to label phagocytic cells having access to blood-borne particles using FITC-labelled *pseudomonas* bacteria injected in the jugular vein. Peripheral blood mononuclear cells (PBMC), broncho-alveolar lavage (BAL) and parenchyma were collected 10 minutes post-bacterial injection, cell suspensions were recovered and stained for cDC1, cDC2, moDC and AM/AM-like discrimination as previously described^4^ (Fig. 1a). In the parenchyma, 19% +/− 4% of AM-like cells presented a clear phagocytosis of FITC-labelled bacteria, no cDC1 and few cDC2 were FITC-stained. Interestingly, moDC presented a highly variable FITC staining, from 2% to 18% (mean 13% +/− 5%). In the BAL, no FITC staining either of AM (Fig. 1a,b) or of BAL DC (data not shown) was observed. In order to identify blood-circulating cells that might phagocytose bacteria and contaminate the parenchymal preparation, the same gating as for parenchymal cells was applied for PBMC. No events were observed that could correspond to AM-like cells (empty gate 4 in Fig. 1a), proving that AM-like cells were not blood circulating cells. Conversely, the PBMC gates 2 and 3 (Fig. 1a), corresponding respectively to cDC2 and moDC in the parenchymal gating, presented a strong phagocytosis of bacteria, since around 50% of these two cell types presented FITC staining. Thus, the FITC staining of parenchymal cDC2 and moDC might mostly be due to blood circulating cell contamination of the parenchyma. In order to demonstrate that the lower staining of AM, cDC1 and cDC2 was not due to an intrinsic defect in bacterial phagocytosis, BAL cells were incubated *in vitro* with FITC-stained pseudomonas. All the DC/Macrophages subtypes (cDC1, cDC2, moDC and AM) presented more than 45% bacteria phagocytosis (data not shown), in agreement with similar capacities of these cells to phagocyte bacteria, provided that they enter in contact with the pathogen.

Thus, AM-like cells are the only parenchymal residing MNP population consistently phagocytosing blood bacteria. Although only 20% of the AM-like cells appeared FITC positive, we consider that the 3 to 4 hours needed for MNP extraction from lung tissue allowed a majority of PIM that had phagocytosed bacteria to quench the acid-sensitive FITC, eventually exhibiting no staining. Alternatively, PIM might be able to move across the blood barrier and locate temporarily in the interstitium, losing their access to blood. We then proceeded to tissue staining and confocal imaging in order to visualize blood-borne bacterial phagocytosis *in situ*. We observed the localization of FITC-stained bacteria inside CD163^high^/MHC-II^high^ cells (AM-like cells) in the parenchyma, in ve-Cadherin (a blood vessel specific marker^21^) rich area (Fig. 1c). Altogether, we show here that AM-like cells are *bona fide* PIM.

### PIM/AM-like cells share with AM differential expression of key chemokine receptors and transcriptional regulators suggesting that they could be derived from the same embryonic precursors

**Figure 2:**
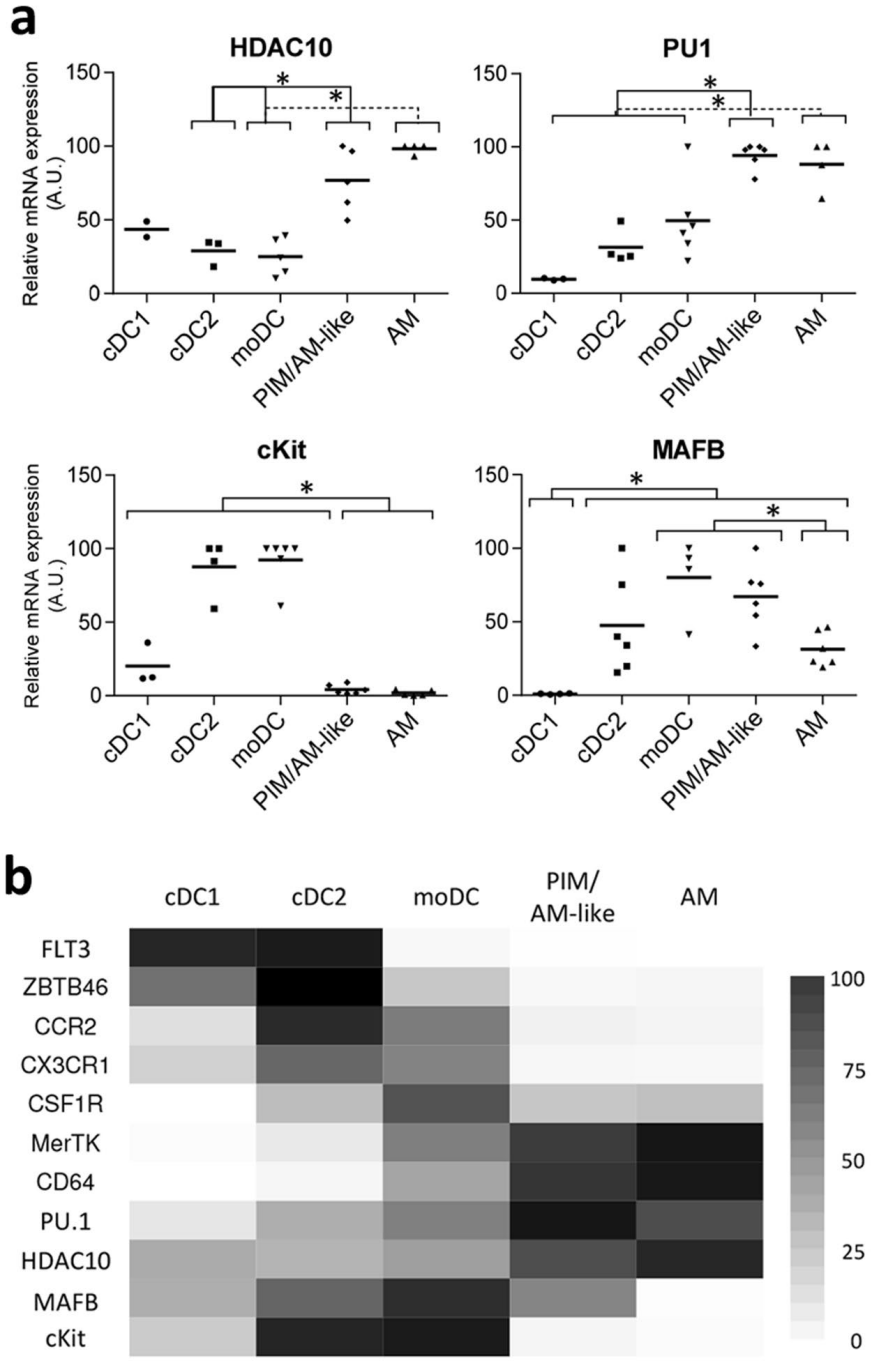
PIM/AM-like cells and AM present a gene expression profile characteristic of embryonically-derived macrophages. Conventional DC1, cDC2, moDC, PIM/AM-like cells and AM were gated as in Fig. 1 and sorted by flow cytometry. (**a**) mRNA expression levels of embryonic-monocytes derived Mϕ associated genes (HDAC10, PU1) and bone marrow precursors derived Mϕ-associated gene (cKit); as well as of MAFB, assessed by RT-qPCR. For each gene, data were normalized to the reference gene RPS24 (ribosomal protein S24) expression and presented as relative expression (arbitrary units (AUs)): for each animal, the population with the highest expression for this gene was considered as 100 and the other populations were normalized to it. Each symbol represents one animal. Statistics: Mann-Whitney test *p < 0.05. (**b**) Heat map synthesizing the means of real-time PCR data from A and from Maisonnasse *et al*.^4,5^.

Our previous work showed that AM and PIM/AM-like cells were very similar, for instance they did not express CCR2 and CX3CR1, unlike the other lung DC/Macrophages populations, whereas they both expressed high levels of MertK and CD64^4^. We then tested the differential expression of genes between macrophages derived from blood monocytes or from tissue resident embryonically-derived, self-renewable macrophages as described by Geissmann and Lambrecht teams^22,23^. Both AM and PIM/AM-like cells strongly expressed the markers differentially represented in embryonic-derived macrophages, HDAC10 and PU.1, whereas they expressed significantly lower amount of the hematopoietic cell marker c-Kit (Fig. 2a,b). This transcriptomic profile was highly distinctive from blood monocytes^24,25^ and definitely excluded the possibility that PIM/AM-like cells were sequestered lung monocytes. Interestingly, MAFB, a gene involved in the decrease of macrophage proliferation during their differentiation^26^, was the only observed marker differentially expressed in PIM/AM-like cells and AM. The PIM/AM-like cells express higher levels than AM (Fig. 2). Like AM, porcine PIM/AM-like cells are likely embryonic-derived macrophages, whose renewal must be independent from blood monocytes.

### PIM/AM-like cells are infected, produce PRRSV and are pro-inflammatory upon *in vitro* infection

**Figure 3:**
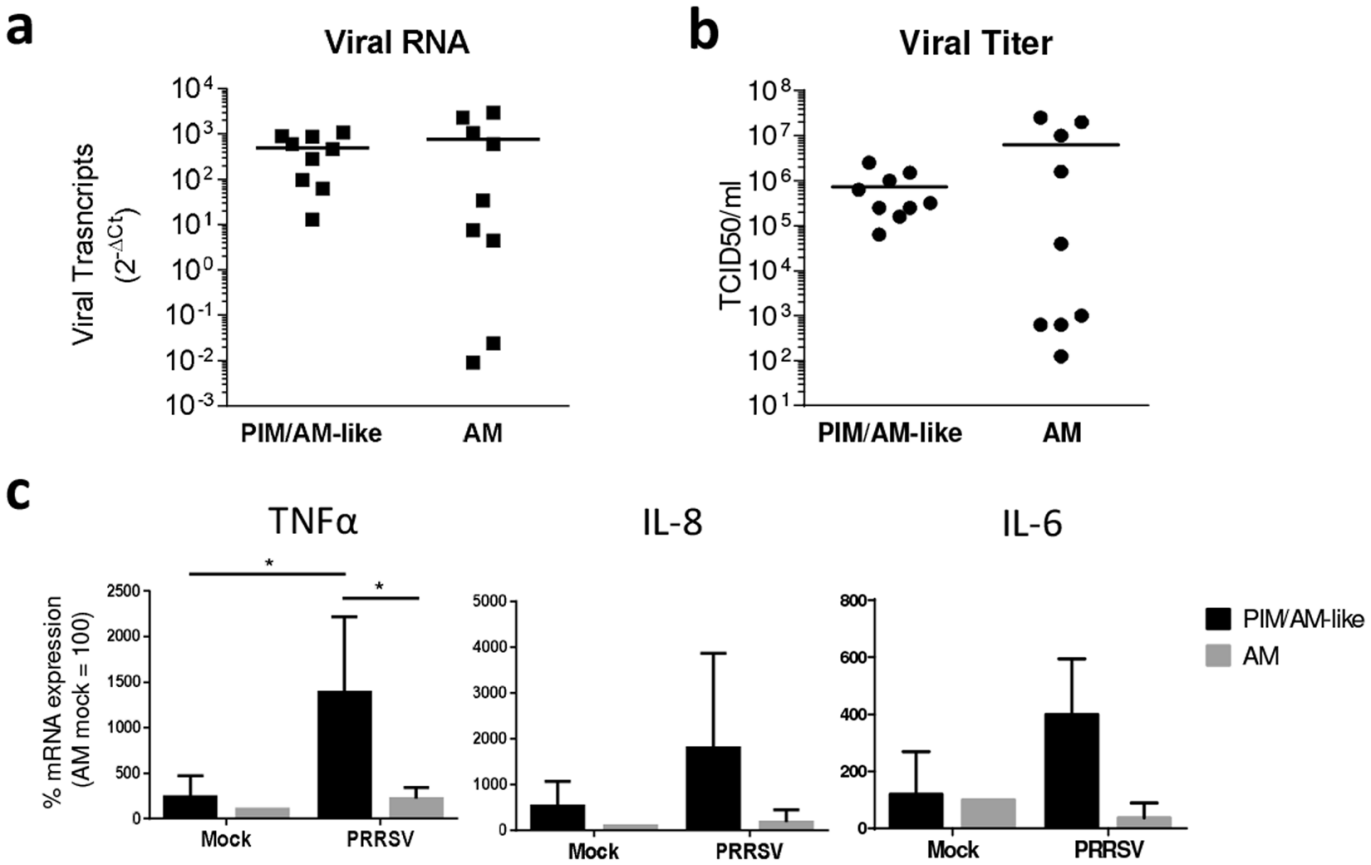
PIM/AM-like cells produce virus and upregulate inflammatory cytokines expressions upon PRRSV infection *in vitro*. (**a** and **b)** Cell-sorted PIM/AM-like cells and AM were *in vitro* infected at 10^–3^ MOI with LENA PRRSV strain. After 24h infection, the cell pellet and the supernatant were collected. (**a**) mRNA were extracted from cell pellets, retrotranscripted and virus RNA contents was quantified by qPCR (n = 9). (**b**) Infectious viral particles (TCID50/ml) were titrated in the supernatant according to the OIE manual (n = 9). Mock infected cells gave no signal in (**a)** as in (**b**) (data not shown). In (**c)** Cell-sorted PIM/AM-like cells and AM were *in vitro* infected at 5 MOI, cell pellet mRNA were extracted, retrotranscripted and the contents in TNFα, IL-8 and IL-6 transcripts were quantified by qPCR (n = 4, (**c**). For each animal, mock infected AM 2^−ΔCt^ mRNA cytokines expression (normalized to the reference gene RPS24) is considered as reference 100, from which PRRSV AM as well as mock and PRRSV AM-like/PIM conditions are normalized. Statistics: Mann-Whitney test *p < 0.05.

Because of the great similarities between AM and PIM/AM-like cells and since AM are thought to be the main target of PRRSV, we compared the susceptibility of AM and PIM/AM-like cells to PRRSV infection using the genotype 1.3 LENA virus^27^. AM and PIM/AM-like cells were cell-sorted and infected *in vitro* at low multiplicity of infection (MOI) 10^–3^. Then, 24 h later, intracellular viral RNA load and infectious viral particle titers were measured using RT-qPCR on the cell pellet and virus titration on cell culture supernatant. PIM/AM-like cells and AM show similar PRRSV RNA load (Fig. 3a) and PRRSV infectious particles production (Fig. 3b). However, AM presented a more variable susceptibility to *in vitro* PRRSV infection than PIM/AM-like cells. We repeated the same experiment using a high MOI in order to infect simultaneously a majority of macrophages; we measured the transcriptomic cytokine response of the cells, focusing on TFNα, IL-8 and IL-6 cytokines previously described as highly expressed in PIM^18–20^. PIM/AM-like cells significantly upregulated TNFα expression upon infection, whereas IL-8 and IL-6 were also upregulated, although non-significantly, highlighting the pro-inflammatory role of PIM/AM-like cells upon PRRSV infection (Fig. 3c).

### *In vivo*, PRRSV infection of PIM/AM-like cells correlates with lung and blood PRRSV titer

**Figure 4:**
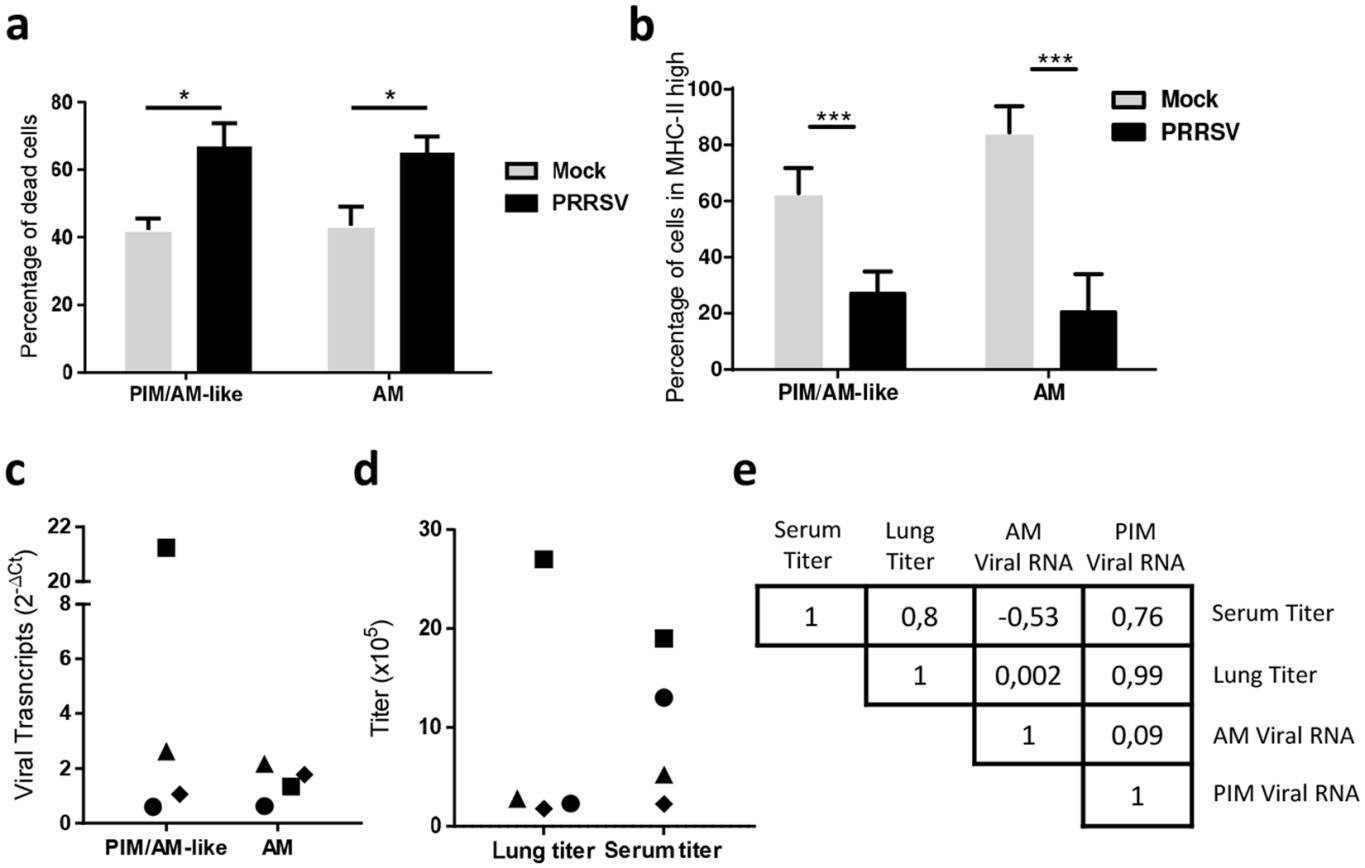
PIM/AM-like cells are depleted upon *in vivo* PRRSV infection and their infection is correlated with viremia and lung titer. *In vivo* infected pigs were euthanized 10 days post-infection, their lung sampled and BAL and parenchymal cells collected, stained, analysed and sorted as above. (**a)** Percentage of dead cells (DAPI-positive) among PIM/AM-like cells and AM, in control (Grey bars, 4 animals) and infected (Black bars, 4 animals) lung after immunostaining. Statistics: Mann-Whitneytest *p < 0.05. (**b**) Percentage of AM and PIM/AM-like cells were monitored in control (Grey bars, 4 animals) and infected (Black bars, 4 animals) lung after immunostaining. Data are expressed as a percentage of MHC-IIhigh cells. Statistics: Mann-Whitney test ***p < 0.001. (**c**) Detection of viral transcripts on sorted AM and PIM/AM-like cells was assessed by RT-qPCR. Data were normalized to the reference gene RPS24 expression. Statistics: Mann-Whitney *p < 0.05. (**d**) Right, lung and serum titers from data previously presented in Renson *et al*.^29^ (**e**) Pearson correlation coefficient determinations of the different parameters obtained from the *in vivo* infection (**c** and **d** data).

Our *in vitro* data prompted us to measure the infection of PIM/AM-like cells during *in vivo* PRRSV infection. We chose to measure AM and PIM/AM-like cells PRRSV infection at an intermediate time point of 10 days post-infection (dpi), at which LENA viremia had reach a plateau^28^. We first measured the percentage of dead cells in PIM/AM-like cells and AM populations. At day 10 post PRRSV infection, we observed a significant increase in the percentage of dead cells of these two populations (Fig. 4a) as previously described for AM^28^. Moreover, in agreement with this result, the proportion of AM and PIM/AM-like cells among MHC-II^high^ BAL and parenchymal cells respectively decrease (Fig. 4b). The amount of PRRS viral RNA was then measured by RT-qPCR on sorted cells (Fig. 4c). Both AM and PIM/AM-like cells were positive for viral RNA. A correlation analysis was performed with the following parameters: AM and PIM/AM-like cells PRRSV infection titers (Fig. 4c), serum PRRSV titers (viremia, as depicted in^29^) as well as whole lung tissue viral load (Fig. 4d). PIM, but not AM infection, appeared correlated with lung titer (0.99 and 0.0025 respectively), and with serum titer (0.76 and −0.53 respectively) (Fig. 4e). This correlation must be taken with caution because it mainly stands on one individual presenting high viral titer in blood, lung and PIM/AM-like cells but not in AM.

**Figure 5:**
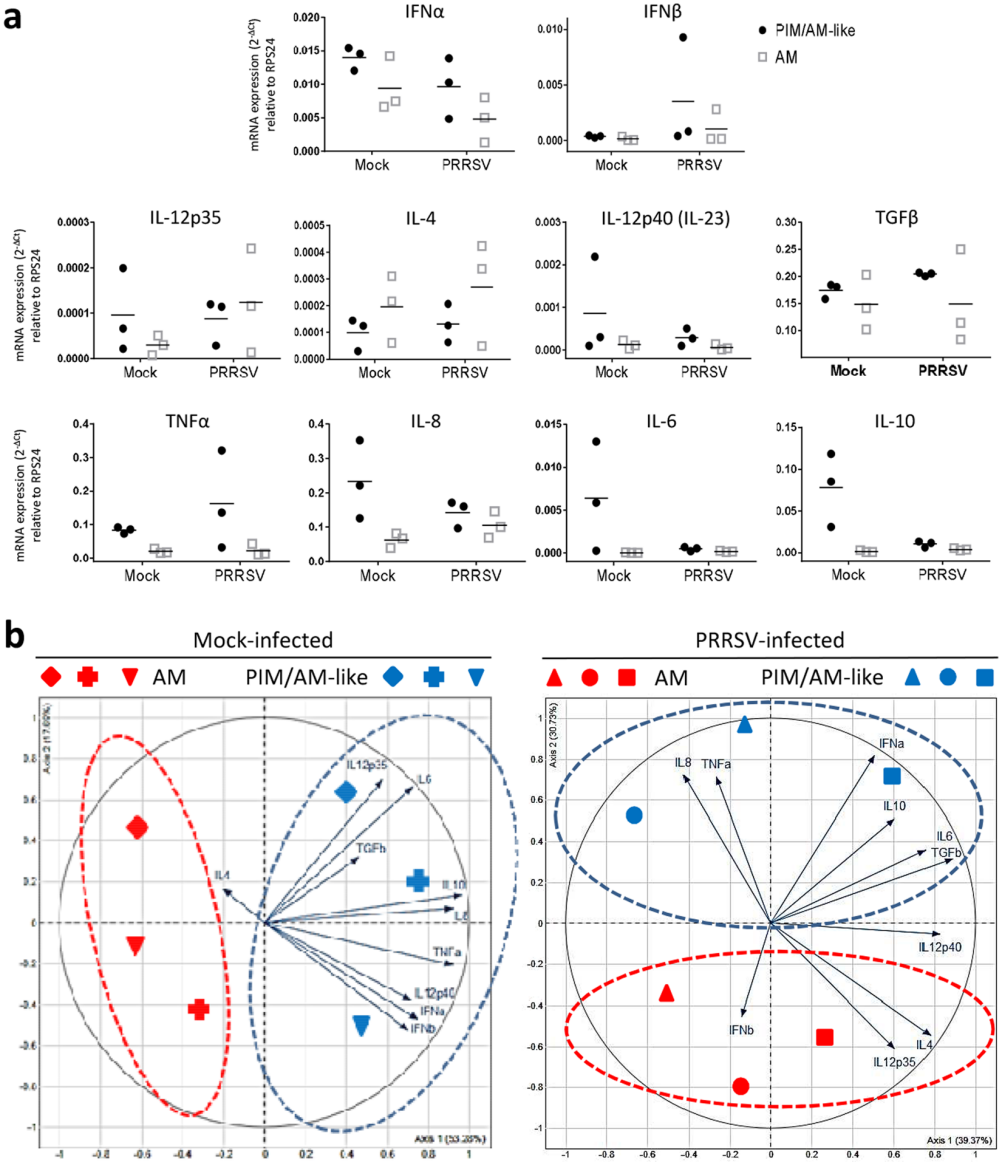
PIM/AM-like cells produce more cytokines than AM at steady state and upon PRRSV infection *in vivo*. *In vivo* infected pigs were euthanized 10 days post-infection, their lung collected and BAL and parenchymal cells extracted, stained, analysed and sorted as above. **(a)** Transcriptomic expression (RT-qPCR) of different cytokines of the innate and adaptive immune response. For each animal, mock infected AM 2^−ΔCt^ mRNA cytokines expression (normalized to the reference gene RPS24) is considered as reference 100, from which PRRSV AM as well as mock and PRRSV AM-like/PIM conditions are normalized. (**b)** Principal Component Analysis of the cytokines transcriptomic expressions raw data (2^−ΔCt^) for AM and PIM/AM-like cells of the 3 mock infected (left) and 3 PRRSV infected (right) animals depicted in (**a)**. Each symbol represents the projection on the PCA analysis of one of the 6 different animals cell types (AM in red and PIM/AM-like cells in blue).

We finally measured by RT-qPCR, the *in vivo* cytokine response of AM and PIM/AM-like cells in mock and PRRSV-infected animals at 10 dpi. No cytokine presented a significant differential expression when conditions or cell types were compared (Fig. 5a). In order to assess the global AM/PIM responses, we then ran a principal component analysis (PCA) on the cytokine data, comparing AM and PIM/AM-like cells in non-infected or infected conditions. PIM/AM-like cells produced more cytokine mRNA than AM except for IL-4 in mock infected animals, and for IFNβ, IL-12p35, IL-12p40/IL-23 and IL-4 in infected animals. In infected animals, the first axis of the PCA encompassed 39% of the total variability of the samples (Fig. 5b). It segregated IL-8 and TNFα from IFNα, IL-6, TGFβ, IL-4, IL-12p35 and IL-12p40/IL23 cytokines. Except for the inter-individual variations, no obvious interpretation for this splitting can be proposed. Interestingly the second axis still encompassed 30% of the variability and clearly segregated PIM/AM-like cells from AM. Indeed PIM/AM-like cells expressed more IL-8, TNFα, IL-6 and IFNα, whereas AM expressed more IL-12p35 and IL-4. Thus we could observe *in vivo*, a tendency of PIM to produce more cytokines than AM at steady state and upon infection. However globally, at 10dpi, both PIM/AM-like cells and AM responded weakly to PRRSV.

## Discussion

Herein, we showed that the previously described porcine AM-like cells^4^ phagocytosed blood-borne bacteria and produced inflammatory cytokines such as TNFα, IL-8 and IL-6^18^. Some teams succeeded in isolating PIM using perfusion of the pulmonary vasculature with collagenase solution^30^. Using this technique, the best controlled experiment^31^ measured a PIM/AM ratio of 1:10, in perfect agreement with the AM-like/AM ratio we previously observed^5^. Despite our inability to stain all AM-like cells, this last data associated with the homogeneity of AM-like cell population phenotype^4^ are in strong support with the whole AM-like cell population being *bona fide* PIM.

Thanawongnuwech observed 20 years ago that collagenase-isolated PIM could be infected by PRRSV^31^, moreover *in vivo* PRRSV infection decreased the ability of the lung to retain copper particles^32^, in agreement with a PRRSV-mediated PIM depletion. We demonstrated here that AM-like cells were as permissive as AM to PRRSV infection *in vitro* and *in vivo*. Thus viral replication is at least as high in PIM/AM-like cells than in AM, demonstrating that AM are not the sole source of virus in the lung. Moreover, because of PIM/AM-like cells localization in the blood vessel lumen, allowing them to shed virus directly in the blood circulation, they might likely be the major cell types responsible for viremia.

Interestingly, we also showed here that both porcine AM and PIM/AM-like cells harbored characteristics suggesting that they derive from embryonic monocytes rather than from adult bone marrow, in consistency with what has been demonstrated for mouse AM^22,23^. Although surprising according to their location in contact with blood, PIM origin can be paralleled with the recently described resident arterial macrophages in mice^33^. These cells are embryonic monocyte-derived, and self-renew in the aortic wall^33^. It has been shown that PIM had an important role in xenogeneic graft failure, and that their depletion before pig lung graft to primate extended the duration of graft functionality^34^. The embryonic origin of PIM implies that the lung vasculature wall of the PIM-depleted-graft can be re-populated from progenitors present within the graft. Thus, the detrimental effect of PIM in the context of xenograft might only be postponed by PIM depletion before grafting.

PIM have been described in the species from the *Laurasiatheria* superorder whereas no PIM were observed in primates and mice, species belonging to the *Euarchontoglires* superorder^6,7,9,14^. However, in rats and humans, evidence exist of a few constitutive PIM that increase in number upon liver dysfunctions^35–37^. In the rat model, PIM induced upon bile-duct ligation were shown to be responsible for endotoxin-induced mortality^38^. It would thus be interesting to re-examine the role of PIM in the liver-induced inflammatory medical conditions.

Interestingly, it has been shown in lambs that PIM and AM appeared in the developing lung within the same time frame, increasing progressively from 1 day to 3 weeks of age^39^. It is tempting to speculate that AM and PIM originate from the same lung cell precursor that relocate after birth either in the alveoli or the lung endothelium. This precursor would have, even in *Euarchontoglires*, the potential to differentiate in PIM, provided the appropriate, still unknown, endothelial/blood stimulus is present.

It is worth noting that AM express lower MAFB mRNA than PIM/AM-like cells. MAFB has been described as a repressor of cell-renewal capacities in adult macrophages^40^. Thus the higher MAFB expression in PIM/AM-like cells would be in agreement with a lower proliferation capacity compare to AM, raising the interesting possibility that PIM/AM-like cells would originate from AM.

## Materials and Methods

### Pseudomonas FITC-staining

1.10^10^ cfu of UV-inactivated *pseudomonas* were resuspended at 2 mg/ml in 0.1 M Na_2_CO_3_ pH9 buffer and 150 µg of fluorescein isothiocyanate (FITC) (SIGMA) per ml of bacteria were added and incubated for 2h at 4 °C. Bacteria (FITC-*pseudomonas*) were then washed twice in PBS and frozen in glycerol before use.

### Pig lung cells collection

For *in vitro* characterization and infection, lung tissue samples were obtained from 5- to 7-month-old Large White conventionally bred sows from UEPAO, Tours, France. A BAL procedure was performed twice on the isolated left lung with 250 ml of PBS supplemented with 2mM EDTA (PBS/EDTA), to collect AM. Next, a 1-cm slice of external lung parenchyma was dissected from the same lung. Tissues were minced and incubated in nonculture-treated Petri dishes, to avoid differential plastic adherence of MΦ and DC, for 2 h at 37 °C in complete RPMI, consisting of RPMI 1640 supplemented with 100 IU/ml penicillin, 100 mg/ml streptomycin, 2 mM L-glutamine, and 10% inactivated foetal calf serum (FCS) (all from Invitrogen, Paisley, UK), containing 2 mg/ml collagenase D (Roche, Meylan, France), 1 mg/ml dispase (Invitrogen), and 0.1 mg/ml Dnase I (Roche). Cells were passed through 40 µm cell strainers and red blood cells lysed with erythrocytes lysis buffer (10 mM NaHCO_3_, 155 mM NH4Cl, and 10 mM EDTA). Next, cells were washed with PBS/EDTA, counted, and step-frozen in FCS/10% dimethyl sulfoxide (Sigma-Aldrich, St Louis, MO) before staining and flow cytometry analysis or cell sorting.

For *in vivo* PIM targeting, experiments were performed at INRA GABI (Jouy en Josas, France). The animal experiment protocol was approved by the French Ethics Committee for Animal Experiments (Comité d’Ethique en Expérimentation Animale du Centre INRA de Jouy-en-Josas et AgroParisTech) and authorized by the French Ministry for Research (authorization no. 2017051200952846). All methods were performed in accordance with the relevant guidelines and regulations. The right jugular vein of 30 to 40 weeks old Melanoma Libechov Minipigs^41^ conventionally bred from GABI (INRA, Jouy en Josas, France) were catheterized under gaseous anaesthesia in order to reach the right atrium. Before FITC-*pseudomonas* injection, 30 ml of blood was withdrawn in heparinized tube as negative control. 0.6 to 10 mg of FITC-*pseudomonas* were resuspended in 5 ml physiological serum and injected in the catheter. After 10 minutes, 30 ml of blood was withdraw in heparinized tube as positive control. The animal was euthanized and the lung was sampled. An extensive BAL was processed using 3 times 100 ml PBS/EDTA. Parenchyma was collected, a 1 cm piece was frozen in Tissue Teck (Sakura, Paris, France) and conserved at −80 °C, and the remaining tissue was minced and digested in Collagenase/Dispase/DNase medium, and single cell suspension was retrieved as previously described^4^. BAL and parenchymal cell suspensions were then frozen in FCS/10% DMSO. Blood cells were ficolled and PBMC were then frozen in FCS/10% DMSO.

### Flow cytometry analysis and cell sorting

Pig cell surface staining was performed as previously described^4^, using anti-MHC-II (clone MSA3) and anti-CD172a (clone 74-22-15a) (monoclonal antibody center Washington State University - Pullmann, WA), unlabelled or Phycoerythrin (PE) coupled anti-CD163 (clone 2A10/11) from Serotec (Oxford, UK) and isotype matched secondary antibodies coupled to PE, Alexa-488, and Alexa-647 (Invitrogen). Infected cells were then fixed in 4% paraformaldehyde before flow cytometry analysis. Samples were acquired on a Fortessa (BD-Bioscience) or sorted on a MoFlo ASTRIOS (Beckman-Coulter). For sorting, preparations were enriched in DC/Macrophages by gradient (Optiprep; Nycomed Pharma). Acquired data were analysed using FlowJo software (version X.0.6).

### Microscopy

Sections of 14 µm were obtained from the parenchyma frozen in Tissue Teck using a cryostat (Leica CM3050S, Nanterre, France) and deposed on Superfrost® glass slides (ThermoFisher scientific). Cryosections were fixed in methanol/acetone (1:1) at −20 °C for 20 min. Fixed slides were saturated using PBS supplemented with 5% horse serum (HS) and 5% swine serum (SS) 30 min at room temperature (RT). The same primary antibodies anti-MHC-II, anti-CD163 as in flow cytometry were used as well as anti-FITC (Fluorescein/Oregon Green Polyclonal Antibody, Alexa Fluor 488, ThermoFisher scientific) and a poly-clonal rabbit anti-ve-Cadherin (H-72, Santa-Cruz Biotechnology, UK), and secondary antibodies were added at 4 °C overnight or 30 min, respectively. Slides were analyzed using an LSM510/U700 confocal microscope (Zeiss, LePecq, France).

### Cell infection and viral titration

Sorted populations were cultured in complete RPMI for 24 h in flat-bottom 96-well plates with 3.10^5^ cells/well and then infected at a MOI of 10^−3^ with a PRRSV virus (LENA) in complete RPMI. At 24hpi, supernatants were collected and frozen at −80 °C. After washing, cells were lysed in 100µL of RNA extraction buffer from the Arcturus PicoPure RNA Isolation kit (Life Technologies) for quantification of viral RNA by RT-qPCR. The same protocol was applied with a MOI of 5 for quantification of cytokine production by RT-qPCR. PRRSV titers (TCID50/ml) were assessed according to the OIE manual of diagnostic test.

### RNA extraction

Total RNA from sorted cells or infected cells were extracted using the Arcturus PicoPure RNA Isolation kit according to the manufacturer’s instructions. Contaminating genomic DNA was removed using a Qiagen RNase free DNase set.

### Real-time quantitative PCR (qPCR)

RNA was reverse transcribed using random hexamers and the Multiscribe reverse transcriptase (Life Technologies). qPCR were performed as previously described^4^. The primers used were: HDAC10 (F: CCGCCGCCAATGGAT, R: GGGCATGCTTGGCTGCTA), PU1 (F: TCCCCCCTCAGCCATCA, R: GCGTTTGGCGTTGGTAGAGA), cKit (F: TGGGCTCGAGAAGTCAAGTATTT, R: ATGCCCGGAGAGCATTTTT), MAFB (F: TGCGTTCTTTAGACCAATATGTTATGT, R: CACCAATAACTCGCCCGCTAT), PRRSV (F: ATGGCCAGCCAGTCAATCAG, R: GGAACGTTCAGTTCCGGTGA); TNFα (F: TGGTGGTGCCGACAGATG, R: CAGCCTTGGCCCCTGAA); IL-6 (F: CTGCTTCTGGTGATGGCTACTG; R: GGCATCACCTTTGGCATCTT). IL-8. 8 (F: TCCTGCTTTCTGCAGCTCTCT, R: GCACTGGCATCGAAGTTCTG); IFNα (F: TCTGCAAGGTTCCCAATGG, R: GGCATTGCAGCTGAGTAGCA); IFNβ (F: CAGCAATTTGGCATGTCAGAA, R: TTCATCCTATCTTCGAGGCAATATT); IL-12p35 (F: CGTGCCTCGGGCAATTATAA, R: CAGGTGAGGTCGCTAGTTTGG); IL-4 (F: GCCGGGCCTCGACTGT, R: TCCGCTCAGGAGGCTCTTC); IL-12p40/IL-23 (F: GGAGCACCCCACATTCCTACT, R: TTCTCTTTTGTTCTTGCCCTGAA); TGFβ (F: GAAGCGCATCGAGGCCATTC, R: GGCTCCGGTTCGACACTTTC); IL-10 (F: GAGCCAACTGCAGCTTCCA, R: TCAGGACAAATAGCCCACTAGCTT). For each gene, data were normalized to the reference gene RPS24 (Ribosomal Protein S24) expression and presented as relative expression (arbitrary units (AU)). In Fig. 2, for each animal, the population with the highest expression was considered as 100 and the other populations were normalized to it. Each symbol represents one animal.

### *In vivo* infection and tissue collection

For *in vivo* PRRSV infections, experiments were performed at ANSES (Ploufragan, France). The animal experiment was authorized by the French Ministry for Research (authorization no. 2015060113297443) and approved by the national ethics committee (authorization no. 07/07/15). Eight specific pathogen-free (SPF) pigs (free from PRRSV, *Actinobacillus pleuropneumoniae, Mycoplasma hyopneumoniae)* were housed in biosecurity level-3 air-filtered animal facilities. Treatments, housing, and husbandry conditions conformed to the European Union Guidelines (Directive 2010/63/EU on the protection of animals used for scientific purposes). At 10 weeks of age, 4 pigs were inoculated intranasally with LENA strain (5.10^5^ TCID50/per animal in 2.5 ml per nostril). Ten days post infection, animals were anesthetized (Zoletil, Virbac, France) and exsanguinated. BAL cells were collected using 500 ml PBS/EDTA for right and left diaphragmatic lobes. BAL and parenchymal cells were sampled as above (pig lung cells collection). Cells were washed in PBS/EDTA and frozen in FBS/10% DMSO. A part of the data issued from this *in vivo* infection has already been published in^28^.

### Statistical analysis

All data were analysed using Graph Pad Prism (version 6) for Mann-Whitney test; R software (version 3.4.0) and package FactoMineR (version 1.39) for *Principal Component Analysis* method (PCA) and Pearson correlation coefficient determinations. When scatter plots are used, the mean is depicted by a horizontal bar.

### Data availability

The datasets generated during and/or analysed during the current study are available from the corresponding author on reasonable request.
